# COVID-19 Pandemic and Osteoporosis in Elderly Patients

**DOI:** 10.14336/AD.2021.1201

**Published:** 2022-07-11

**Authors:** Jun Tang

**Affiliations:** Department of Endocrinology, Zhongnan Hospital of Wuhan University, Wuhan, China.

**Keywords:** COVID-19, osteoporosis, aging, glucocorticoid

## Abstract

Coronavirus disease 2019 (COVID-19), which is caused by an infection of severe acute respiratory syndrome coronavirus 2 (SARS-CoV-2), is rapidly becoming a worldwide epidemic and poses a significant threat to human life and health. SARS-CoV-2 can cause damage to organs throughout the body through ACE2 receptors. It may have direct and indirect effects on osteoclasts, and osteoblasts and lead to osteoporosis. Vitamin D (VitD) is a key hormone for bone health and has immunomodulatory actions of relevance in the context of the COVID-19 pandemic. Vitamin D deficiency has a significant positive association with both infection and the mortality rate of COVID-19. Elderly patients infected by COVID-19 were more likely to develop acute respiratory distress syndrome (ARDS), which was primarily caused by an inflammation storm. The production of proinflammatory cytokines increases with COVID-19 infection and immobilization may result in bone loss and bone resorption in seriously ill patients, especially aging patients. It is well known that glucocorticoids are beneficial in the treatment of acute respiratory distress syndrome (ARDS) because they reduce inflammation and improve the functioning of the lung and extrapulmonary organs. Glucocorticoid therapy is widely used to treat patients with COVID-19 in most parts of the world. During COVID-19 clinical treatment, glucocorticoids may accelerate bone loss in elderly people, making them more susceptible to the development of osteoporosis. Therefore, it is worthwhile to draw the attention of clinicians and researchers to the linkages and interactions between COVID-19, glucocorticoids, and osteoporosis (especially in elderly patients).

Coronavirus disease 2019 (COVID-19) is caused by severe acute respiratory syndrome coronavirus 2 (SARS-CoV-2) infection and develops into pneumonia [1]. The number of cases in some regions is still growing because of resource-constrained health systems and a high prevalence of chronic conditions.

Following the effective control of the epidemic in the most countries and the gradual transition into the post-epidemic era, clinical observation of recovering patients is also at the forefront. Vitamin D (VitD) is a key hormone for bone health, and several studies have found that people with vitamin D deficiency are more likely to develop COVID-19 [2-4]. In the pandemic, most critically ill patients are aging [5] and are at high risk of osteoporosis, and some of them have received glucocorticoid (GC) therapy [6]. The symptoms of glucocorticoid-induced osteoporosis (GIOP) are insidious and not easily detected in the early stage, thus, a series of complications such as fracture and osteonecrosis may occur later. If glucocorticoid therapy is not promptly investigated and treated, the development of osteoporosis may be further aggravated, so the impact of GC therapy on bone metabolism must also be considered.

However, in the current epidemic, few studies have focused on osteoporosis and other bone metabolic diseases, and current studies related to osteoporosis mainly focus on how to prevent and standardize treatment [7,8], but the type of influence on bone metabolism such as osteoporosis in COVID-19 patients has not been deeply studied. Therefore, this article provides a review of COVID-19 infection, glucocorticoid therapy, and possible contributionsto osteoporosis.

## Influence of SARS-CoV-2 on osteoporosis (OP)

Several factors influence SARS-CoV-2's impact on the human body, and it has been found that most of this virus's effect can be attributed to binding to the angiotensin-converting enzyme 2 receptor (ACE2) [9]. The expressionof ACE2 is highest in adipose tissue, testis, kidney, heart, thyroid and small intestine [9]. There is no significant difference in the expression of ACE2 between men and women, or between young and old individuals in any tissue [10].

Several similarities exist between SARS-CoV and SARS-CoV-2, including the spike proteins, which can bind to mACE2. They also possess a receptor-binding domain that is nearly identical in the 3-D structure [11]. The coronavirus that causes SARS (SARS-CoV) causes serious lung disease with a high mortality rate. According to a previous study, Saemi Obitsu et al. found that after SARS infection, several skeletal abnormalities were observed in patients following recovery from SARS, including decreased bone density and osteonecrosis. Some of these findings could be explained by short-term steroid use, but not completely. Therefore, they found that human mononuclear cells, namely, osteoclast precursors, could partially express ACE2 [12].

By expressing SARS-CoV accessory protein 3a/X1 in the murine macrophage cell line RAW264.7 differentiation into osteoclast-like cells was found to be enhanced through stimulation with receptor activator of nuclear factor-κB ligand (RANKL). Overexpression of 3a/X1 in human epithelial A549 cells increased the expression of tumor necrosis factor-alpha (TNF-α), which is known to accelerate osteoclastogenesis, and 3a/X1 also increased RANKL expression in mouse stromal ST2 cells. These results indicate that SARS-CoV accessory protein 3a/X1 directly or indirectly promotes osteoclastogenesis, thereby accelerating bone resorption and affecting bone density [12].

Short-term effects of SARS-CoV-2 on skeletal tissue have been studied. Olatundun D et al. demonstrated that within 2 weeks postinfection, SARS-CoV-2 dramatically increases osteoclastogenesis and results in bone loss in a COVID-19 mouse model [13].

Bobin Mi et al. found that IgG^+^patients exhibited the greatest differential expression of miR-4485-3p. MiR-4485-5p has been shown to negatively regulate bone remodeling in vitro and in vivo. Researchers discovered that miR-4485 regulation suppresses osteogenic differentiation in COVID-19 patients, and TLR-4 may be the target of miR-4485. Osteogenic differentiation is suppressed by SARS-CoV-2-induced overexpression of miR-4485, providing a promising target for anti-osteoporosis therapy in COVID-19 patients [14].

These studies have shown that SARS-CoV-2 can directly or indirectly act on osteoclasts and osteoblasts, affecting bone metabolism, and leading to osteoporosis, etc. Further research is needed to explore the long-term effects of SARS-CoV-2 infection on skeletal health.

## Association between Vitamin D and COVID-19

Vitamin D (VitD) is a key hormone for bone health and a regulator of immune function, which is relevant in the context of COVID-19 [15]. The risk of osteoporotic fractures is increased in patients with vitamin D deficiency, and many diseases are associated with it. Bone turnover, bone loss, and osteoporotic fractures are accelerated in patients with vitamin D deficiency (serum 25-hydroxyvitamin D <50 nmol/L) [15]. As a facilitator of innate and adaptive immunity, vitamin D may be relevant to COVID-19 [16].

A study conducted by Katz J. et al. found that patients with vitamin D deficiency had a 4.6 times greater chance of testing positive for COVID-19 than patients without deficiency ( *p* < 0.001). Among patients with vitamin D deficiency, the incidence of COVID-19 infection was 5 times greater than that among those without deficiency after age adjustment (OR = 5.155; *p* < 0.001). An increased risk for COVID-19 can be attributed to vitamin D deficiency [2]. Ranil Jayawardena et al. foundthat among Asian countries, vitamin D deficiency positively correlates with COVID-19 infections (r = 0.55; *p*= 0.01; R = 0.31) and mortality (r = 0.50; *p* = 0.01; R = 0.25). A significant negative correlation was seen between mean vitamin D levels and COVID-19 infections (r = -0.77; *p* = 0.04; R = 0.59) and mortalities (r = -0.80; *p* = 0.03; R = 0.63). When confounding variables were taken into account, the prevalence of vitamin D deficiency had a significant positive association. In contrast, the mean vitamin D level had a significant negative association with both the infection and mortality rate of COVID-19 in Asian countries [3]. According to the study of Alberto Sulli et al., 25OH-vitamin D deficiencies in aging COVID-19 patients are linked to more severe lung involvement, longer disease duration, and a higher risk of death. Deficiency of vitamin D was detected in younger COVID-19 patients without comorbidities, suggesting that it is an important risk factor that should be considered by all[4]. In an Israeli population-based study, Eugene Merzon found that COVID-19-positive individuals had lower plasma vitamin D levels than COVID-19-negative individuals. Univariate analysis shows an association between a low plasma 25(OH)D level and an increased risk of SARS-CoV-2 infection, and hospitalization due to COVID-19 infection [17]. According to a prospective study in the UK Biobank, Hao Ma et al. reported that when covariates were adjusted for, the habitual use of vitamin D supplementation resulted in a 34% lower risk of infection (OR, 0.66; 95% CI, 0.45-0.97; *p* = 0.034). Neither the circulating nor genetically predicted vitamin D levels affected the association between vitamin D supplements and COVID-19 infection (P-interactions = 0.75 and 0.74, respectively)[18].

Vitamin D has immunomodulatory properties, including downregulation of proinflammatory cytokines [19,20]. Metabolites of vitamin D do not influence the replication of respiratory viruses or the titers of antibodiesinduced by vaccination, but they do lower cytokine expression induced by a viral infection, including IL6, TNF-α, and IFN-β [20-22]. In addition to these effects, vitamin D also modulates macrophage chemotactic protein 1, interleukin 8, and type 1 interferonand lowers oxygen reactive species [20,23]. By blocking the renin-angiotensin pathway, vitamin D can also attenuate the acute lung injury caused by lipopolysaccharide in mice. Tsujino I et al. recently found that in mice with bleomycin-induced interstitial pneumonia and human cells, vitamin D3 is locally activated in lung tissue, preventing interstitial pneumonia [24]. Although the protective effect of vitamin D on COVID-19 may be related to the inhibition of cytokine responses, a meta-analysis also suggests that regular oral vitamin D2/D3 (2000 IU/d) in patients with vitamin D deficiency has a protective effect against acute respiratory infections [25].

Studies have also been completed to determine whether VITD is effective at treating COVID-19. A randomized, double-blind, placebo-controlled study was conducted in 2 sites in Sao Paulo, Brazil, by Igor H Murai et al. One oral dose of 200 000 IU of vitamin D3 or placebo was randomly administered to patients. In-hospital mortality ( *p*=0.43), admission to the intensive care unit ( *p*=0.30), and mechanical ventilation ( *p*=0.09) were not significantly different between the vitamin D3 group and the placebo group. A high dose of vitamin D3 is not recommended for treating moderate to severe COVID-19 [26]. The study carried out by Chuen Wen Tan et al. determined whether vitamin D, magnesium, and vitamin B12 (DMB) provided clinically significant improvements in the outcomes of older patients with coronavirus disease 2019 (COVID-19). On admission, patients who did not require oxygen therapy received 1000 IU/day vitamin D3, 150 mg/day magnesium, and 500 mcg/day vitamin B12. Combining vitamin D, magnesium, and vitamin B12 has been associated with a significant reduction in oxygen use, intensive care use, or both among older COVID-19 patients [27]. Marta Entrenas Castillo et al. found that through electronic randomization on the day of admission, patients were assigned to take or not take calcifediol (0.532 mg) in a 2 calcifediol to 1 no calcifediol ratio. The patients taking oral calcifediol (0.266 mg) continued this treatment on Day 3 and Day 7. There was one ICU admission out of 50 calcifediol-treated patients (2%), compared to 13 admissions out of 26 untreated patients (50%) ( *p* < 0.001). Throughout the study, there were no deaths or complications with the patients given calcifediol. Those 13 patients who did not receive calcifediol were discharged without being admitted to the intensive care unit. Among the 13 who were admitted to the ICU, two died and the rest were discharged. Their study results indicate that calcifediol or 25-hydroxyvitamin D administration considerably decreased the need for ICU treatment for COVID-19 patients [28]. More importantly, vitamin D is a key point in bone health maintenance, and it is necessary for patients with fragility fractures. In addition, potential beneficial evidence of the immune system, supports the recommendations of achieving optimal vitamin D status in COVID-19 patients [29]. The beneficial role of vitamin D in musculoskeletal health is undisputed. Osteoblasts and osteoclasts are affected by vitamin D, and they can interact with nonskeletal tissues, such as extraosseous tissues and parathyroid hormone (PTH) [30].

Supplementing with vitamin D can prevent falls, according to a previous meta-analysis [31]. Vitamin D along with calcium supplementation is also recommended as a treatment for osteoporosis by the American Society of Bone and Mineral Research [32]. Studies have implicated vitamin D as an adjunctive treatment for osteoporosis and the importance of maintaining bone quality [33]. Reaching an optimal vitamin D status will be beneficial for COVID-19 patients, especially for vitamin D deficient patients, to prevent falls, frailty, and fractures either during or after hospitalization [34-37]. Flavia Tramontana et al. suggested that COVID-19 patients may benefit from achieving optimal vitamin D levels both during and after hospitalization to prevent falls, frailty, and fractures [38]. Combining all these data may indicate that vitamin D not only protects bones but also has potential benefits for the immune system.

## Elderly patients infected with COVID-19 are at high risk for osteoporosis (OP)

Aging was an independent risk factor for death in COVID-19 patients. Age is one of the most important prognostic factors associated with lethality in COVID-19 infection. Studies have shown that the severity and clinical outcome of COVID-19 patients are largely dependent on the age of the patient. Adults over 65 years old accountfor approximately80% of hospitalized patients and the risk of death 23 times higher than that of adults under 65 years old [5].

Aging patients infected by COVID-19 were more likely to develop acute respiratory distress syndrome (ARDS) and cardiac injury than younger patients [39]. This may also be one of the reasons for the high mortality rate among older adults. Additionally, several studies have shown that COVID-19 primarily affects older adults, a population that is at a higher risk for low serum 25-hydroxyvitamin D levels (25(OH)D) [38].

Concerning the in-depth mechanism, inflammasome activation in lung inflammation and fibrosis following SARS-CoV and SARS-CoV-2 infections is mediated by nucleotide-binding domain and leucine-rich repeat family, pyrin domain containing 3 (NLRP3). It is worth mentioning that the NLRP3 inflammasome is overactivated in older adults, and deficient mitochondrial function increases mitochondrial reactive oxygen species (mtROS) and mitochondrial DNA, which results in a hyperresponse from classically activated macrophages and subsequent increases in interleukin-1β (IL-1β) [40].

Early osteoclastogenesis and bone loss are closely related to inflammatory factors [41]. There is no doubt that inflammatory cytokines play an important role in osteoclastogenesis via regulation of the RANK/RANKL/ OPG axis [42]. Furthermore, they stimulate monocytes to express RANK, which prevents bone resorption. In addition, they also inhibit osteoblast formation by restraining OPG. SARS-CoV-2 can probably induce prolonged inflammatorystimuli, which, in turn, will cause the secretion of proinflammatory cytokines if RANKL is not resolved by the homeostatic system [43]. The production of proinflammatory cytokines increases with COVID-19 infection, and this may result in bone loss and bone resorption in seriously ill patients, especially aging patients, who are immobilized for long periods [44].

A prevalence study of low bone mass and osteoporosis among the United States in 2017-2018 indicated that adults aged 50 and over had osteoporosis at either the femur, neck, or lumbar spine at a rate of 12.6%, and those aged 65 and over had a rate of 17.7%, compared to adults aged 50-64 (8.4%). Women aged 65 and over documented the same pattern (27.1% compared with 13.1% among those aged 50-64). The observed age difference among men was not significant (5.7% for 65 and over compared with 3.3% for 50-64). Women had a higher prevalence of osteoporosis than men, regardless of age group [45]. Aged people have a high prevalence of osteoporosis and other bone metabolism diseases. Aging increases the risk of osteoporosis. Additionally, the number of people aged 60 or older has been increasing in many countries over the past few decades [46]. Osteoporotic fractures, particularly hip fractures, which are considered the most serious type, are associated with disability and chronic pain. Loss of independence, decreased quality of life, and death within one year following hip fractures are common in patients with hip fractures [47]. Seventy-one percent of osteoporotic fractures occur among women [48], and the prevalence of osteoporosis in women is generally higher than that in men (15.4% vs. 4.3%, respectively) [49]. Osteoporosis is the leading cause of fractures in postmenopausal women, and more than one-quarter develop vertebral deformities or hip fractures during their lifetimes. Fractures severely affect the quality of life and are becoming a major public health problem owing to the aging population [50]. Ash K Clift et al. conducted a population-based cohort study, and found that women (1.12, 95% CI, 1.00-1.260) and men (1.35 [1.24-1.47]) with hip, spine, humerus, and wrist fractures had a higher mortality rate from SARS-Cov-2 infection. SARS-CoV-2 infection also increased the risk of hospital admission for individuals with previous fractures [51]. Luigi di Filippo et al. performed a retrospective cohort study in Italy. In their study, 114 COVID-19 patients were included, and 41 (36%) were found to have thoracic VFs. Compared with the general population, where VFs are prevalent in 18-26% of women and 8-20% of men. The number of patients requiring noninvasive mechanical ventilation was higher among patients with VFs than among patients without VFs (48.8% vs. 27.4%, *p* = 0.02). In the VF+ group, the mortality rate was 22%, and in the VF- group, it was 10%. Compared with patients with mild and moderate VFs (7% and 24%, respectively), those with severe VFs tended to have higher mortality (60%). According to this study, the presence of VFs was a strong indicator of clinical outcomes and was related to other well-described comorbid conditions [52]. According to the author, VFs may lead to impaired respiratory function and kyphosis, resulting in diminished lung capacity and a greater risk of severe SARS-CoV-2 infection [52]. SARS-CoV-2 infection was found to occur in 1 to 28% of hip fracture patients, with a mean of 13% in one recent meta-analysis [53]. COVID-19-positive patients had a sevenfold higher risk of death than COVID-19-negative patients based on 21 studies that reported mortality following hip fracture. Those with COVID-19 infection died at a rate of 35%, while those without it died at a rate of 8% [53]. As a result of the pandemic, measures such as travel bans, quarantines, and self-isolation led to a reduction in physical activity, particularly in older adults, contributing to sarcopenia and muscle loss [54]. Immobilization also contributes to rapid muscle loss in people 65 years of age and older, as well as an increased chance of falls in seniors caused by COVID-19-related factors as well as chronic inflammation and frailty [55]. People with COVID-19 who are experiencing adverse outcomes should establish a comprehensive recovery plan, which will target their long-term health issues. Osteoporosis anti-treatment and fracture risk assessment may be necessary for older individuals recovering from SARS-CoV-2 [56].

## COVID-19 and glucocorticoid treatment

It is well known that glucocorticoidsare beneficial in the treatment of acute respiratory distress syndrome (ARDS) because they reduce inflammation and improve the functioning of the lung and extrapulmonary organs. Glucocorticoid therapy is widely used to treat patients with COVID-19 in most parts of the world [10,57-59]. Because of their ability to suppress severe systemic inflammation, glucocorticoids are widely used in the treatment of pneumonia and prevention of lung damage. [60,61].

In a controlled, open-label trial, COVID-19 patients were randomly assigned to receive oral or intravenous dexamethasone (6 mg once a day) for up to 10 days or to receive usual care alone. Dexamethasone use decreased 28-day mortality in patients hospitalized with COVID-19 who received either invasive mechanical ventilation or oxygen alone at randomization, but not in those with no respiratory support. Shorter hospital stays (median, 12 days *vs.* 13 days) and a greater likelihood of discharge alive within 28 days (rate ratio, 1.10; 95% CI, 1.03 to 1.17) were found in the dexamethasone group compared with the usual care group. In terms of discharge within 28 days, patients who were receiving invasive mechanical ventilation at the time of randomization experienced the greatest benefit [57]. Bruno M Tomazini et al. conducted a multicenter, randomized, open-label, clinical trial conducted in 41 intensive care units (ICUs) in Brazil. There were three groups of dexamethasone administrations: 20 mg intravenously for five days, 10 mg intravenously for five days or until ICU discharge, plus standard care (n = 151), or standard care alone (n = 148). Dexamethasone-randomized patients had a mean of 6.6 ventilator-free days compared with the standard care group which had a mean of 4.0 days ( *p* = 0.04). The use of intravenous dexamethasone plus standard care was statistically significant among COVID-19 patients with moderate or severe ARDS compared with standard care alone, for ventilation-free days (days alive and without mechanical ventilation) over 28 days [58]. A single-blind, randomized, and controlled trial was conducted in Iran on severely hospitalized patients with confirmed COVID-19 who had been hospitalized since the early pulmonary phase. By using the block randomization method, the patients were randomly assigned to receive standard treatment plus methylprednisolone pulses (intravenous injection, 250 mg/day-1 for 3 days) or standard treatment alone. Researchers found that the methylprednisolone group had a higher rate of improved patients (94.1% versus 57.1%) and the mortality rate was significantly lower than that of the standard-care group (5.9% versus 42.9%; *p*<0.001). The treatment group with methylprednisolone had significantly better survival rates than the control group [59]. A single-center retrospective cohort study was performed by Ana Fernández-Cruz et al. Of these, 396 (46.7%) patients were treated with steroids, while 67 were not. A total of 310 (78.3%) patients received methylprednisolone therapy (22.5% of them were treated with steroid pulses later) and 86 (21.7%) received steroid pulse therapy from the outset. Compared to controls, in-hospital mortality was lower in patients treated with steroids (13.9% [55/396] versus 23.9% [16/67]; *p* = 0.044). As a result of steroid treatment, mortality was reduced by 41.8% compared to mortality without treatment (relative risk reduction, 0.42 [95% confidence interval, 0.048 to 0.65]). According to their findings, patients with SARS-CoV-2 pneumonia who receive glucocorticoids have a better chance of survival than those without glucocorticoids [10].

Osteoporosis and low bone density can result from glucocorticoids adversely affecting bone cell function and mineral metabolism.The majority of bone loss occursduring the first few months after glucocorticoids are given, and even a moderate dosage, which some experts consider to be in the range of physiological replacement, may increase fracture risk [62].

Glucocorticoid-induced osteoporosis (GIOP) is the most common cause of secondary osteoporosis, and it is the third most frequent cause of osteoporosis after postmenopausal osteoporosis and senile osteoporosis [63]. One study found that people taking oral glucocorticoids were nearly three times more likely to suffer a vertebral fracture (2.86, 95% CI 2.56-3.16) and double the risk of hip fracture (2.01, 95% CI 1.74-2.29)[64]. The incidence of fractures depends on both the cumulative dose and the current dose of glucocorticoids. Glucocorticoids were associated with an average fracture incidence (5.1%, 95% CI 2.8-8.2%) in patients initiating treatment (≤6 months use) for vertebral fractures and 2.5% for nonvertebral fractures, When chronic glucocorticoid users (>6 months use) were analyzed, their risks were 3.2% (95% CI 1.8-5.0%) and 3.0% (95% CI 0.8-5.9%), respectively [65]. A current daily dose of <5 mg per day results in an average fracture incidence rate of 9.0 (95% CI 5.7-13.7) per 1,000 person-years, but increases to 16.0 (95% CI 11.0-22.6) at ≥5 mg per day. [66]. An estimated 6-12% of bone mass is lost during the first year of treatment for GC, and this loss slows down to 3% per year thereafter for the long term [67]. In the first 3 months of GC use, bone density begins to decline rapidlyand reaches its peak in the 6th month, and after 1 year, bone loss can be 12%~20% [68]. The effect of GC on bone mass is early and rapid, and then slowly persists, so there is no so-called safe dose or safe mode of administration of GC. GIOP prevention and treatment should be carried out at any time [62]. High-dose glucocorticoids were used to treat severe acute respiratory syndrome (SARS) patients in 2003 [69-73]. E.M.C. Lau et al. found that Hong Kong male patients with SARS had lower hip bone density than normal controls, which may be associated with prolonged hormone therapy [74].

**Figure 1.**
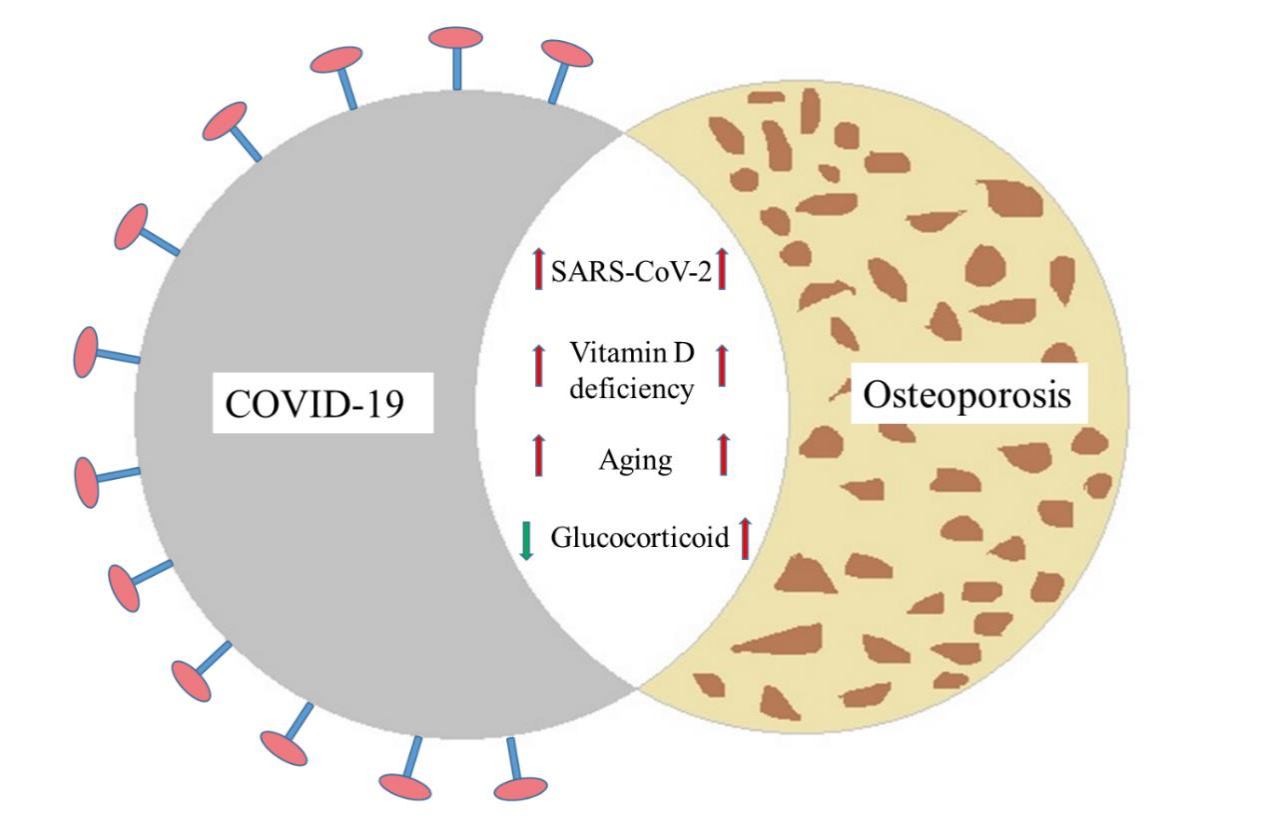
Relation of COVID-19 with osteoporosis.

Similarly, the cumulative dose and duration of treatment with glucocorticoidswere major risk factors for osteonecrosis in SARS patients, according to a meta-analysis of data from 1137 patients [75]. As a consequence of high cumulative doses and longer treatment durations of steroids, avascular osteonecrosis is more common in SARS patients recovering from the illness [75]. Corticosteroid-induced osteonecrosis has affected some survivors of SARS in terms of their quality of life.Michael H M Chan et al. found that SARS patients who were treated with hydrocortisone or methyl-prednisolone at high doses prolonged for an extended period had osteonecrosis [73]. James Francis Griffith found that prednisolone-equivalent doses greater than 3 g caused a 13% risk of osteonecrosis, whereas doses less than 3 g causeda 0.6% risk. In patients receiving steroid therapy for SARS, osteonecrosis was associated with an appreciable dose-related risk [76]. Although femoral head osteonecrosis was frequently observed among patients with SARS who were treated with high-dose glucocorticoids in 2003, a significantly lower cumulative dose and prolonged course of COVID-19 treatment was administered compared to the recommended glucocorticoid doses in SARS treatment. It is therefore prudent to perform risk stratification based on supraphysiologic glucocorticoid dosing provided during the course of COVID-19 treatment and to potentially perform magnetic resonance imaging on those patients with suspected avascular necrosis of the hip [77].Figure 1 demonstrates the proposed relationship of COVID-19 with osteoporosis as described below.

## Management of osteoporosis in COVID-19 pandemic

Sarah J Cromer et al. believed the COVID-19 pandemic decreased screening and disrupted treatment for osteoporosis. During the pandemic, the use of osteoporosis medications is safe and effective and should go on as usual [78]. Salamanna et al. discovered that platelet (PLT) count and monocyte-related factors could be used as biomarkers for screening, diagnosing, and monitoring OP in both sexes at these times [79]. Numerous biological and clinical mechanisms connect bone metabolism and inflammation. A bone metabolism marker (CTX, P1NP, alkaline phosphatase) analysis would be useful in COVID-19 patients [80]. Elaine W Yu et al. recommended that even without BMD testing, stratification of fracture risk could still be carried out using the Fracture Risk Assessment Tool (FRAX) [81]. Atmaca et al. found that the preexisting use of osteoporosis drugs, including bisphosphonate, denosumab, or teriparatide, in women had no effect on COVID-19-related hospitalizations, ICU admissions, or mortality. During a COVID-19 infection, these medications should not be discontinued [82]. Elena Tsourdi et al. suggested that adequate physical activity, vitamin D supplementation, and following a balanced diet are standard nonpharmacologic approaches for bone health; because of their musculoskeletal benefits and potential role as immunocompetence facilitators, these strategies should be maintained [83]. Infection with the COVID-19 virus is not increased or made worse by osteoporosis therapies. In addition, COVID-19 vaccination is not affected by osteoporosis therapies. COVID-19 vaccines do not interfere with osteoporosis therapies, so they should not be halted or delayed if you have received the vaccination for COVID-19. Minor adjustments to the timing of the COVID-19 vaccination schedule may be considered depending on the specific drug profile in an anti-osteoporosis medication category [83]. Patients receiving glucocorticoids are routinely advised to consume adequate calcium (1,000 mg) and vitamin D (600 to 800 IU)[84].

According to the research of Maria Almeida and colleagues, estrogen deficiency during menopause or the loss of both estrogens and androgens in old men leads to osteoporosis, one of the most common and damaging metabolic disorders of old age [85]. Most postmenopausal women with osteoporosis should take the following measures: exercise, avoiding smoking and excess alcohol intake, calcium intake between 1000 and 1500 mg a day, vitamin D intake between 600 and 800 IU a day, and antiresorptive agents. Bisphosphonates are generally recommended as first-line therapy if there are no contraindications [86]. The risk of osteoporosis and fractures, both among males and females who have recovered from SARS-CoV-2 infection, should be investigated in the near future [80].

## Conclusion

With the strengthening of prevention and control measures and the increase in vaccinated populations, COVID-19 has been effectively controlled in many countries and regions, but the impact of this epidemic on human health deserves further attention, especially on bone metabolism such as osteoporosis. For aging COVID-19 patients with vitamin D deficiency who are treated with, glucocorticoids and who are at high risk of osteoporosis, we need to screen and diagnose them and provide prompt treatment, to prevent fractures.
